# Financial Characteristics of Nonprofit Social Welfare Organizations in the US Health Care System

**DOI:** 10.1001/jamahealthforum.2023.1507

**Published:** 2023-06-23

**Authors:** Elizabeth Plummer, Sunjay Letchuman, Shunlan Fang, Ge Bai

**Affiliations:** 1 Neeley School of Business, Texas Christian University, Fort Worth; 2 Burnett School of Medicine, Texas Christian University, Fort Worth; 3 Icahn School of Medicine at Mount Sinai, New York, New York; 4 Ambassador Crawford College of Business and Entrepreneurship, Kent State University, Kent, Ohio; 5 Johns Hopkins Carey Business School, Baltimore, Maryland; 6 Johns Hopkins Bloomberg School of Public Health, Baltimore, Maryland

## Abstract

This cross-sectional study examines the purpose, revenues, profitability, and lobbying expenses of social welfare organizations in the US health care system.

## Introduction

Nonprofit organizations play an important role in the US health care system and receive substantial taxpayer subsidies at the federal, state, and local levels. Prior studies have focused on nonprofit organizations that receive tax-exempt status under Internal Revenue Code §501(c)(3), such as hospitals and charity foundations for prescription drugs.^1,2,3^ To our knowledge, no research has examined health care organizations that are tax exempt under §501(c)(4) (ie, social welfare organizations). To be tax exempt under §501(c)(4), an organization must not be organized for profit and must operate exclusively to promote social welfare. In contrast with §501(c)(3) organizations, which face greater restrictions on permissible activities, §501(c)(4) organizations can engage in a wide range of activities (eg, lobbying), vaguely defined as “promoting social welfare.”^4^ In this cross-sectional study, we examine the purpose, revenues, profitability, and lobbying expenses of social welfare organizations in the US health care system.

## Methods

We used data from all Form 990s (mandatory annual filing) filed by US social welfare organizations during calendar year 2021.^5^ We first identified social welfare organizations that operate in the health care system using organizations’ activity codes from the Internal Revenue Service (IRS) Exempt Organizations Business Master File. We then reviewed each health care social welfare organization, categorized them into groups based on their primary activities, and examined total revenue, profit, and profit margins across categories.

Next, from the US Senate website, we obtained these organizations’ annual lobbying expenses (if any) from 2017 to 2020 (2 preceding election cycles) from required disclosures under the Lobbying Disclosure Act.^6^ We plotted their profits and average annual lobbying expenses relative to revenue. Institutional review board approval was not sought because no human participants were involved. This study was designed and implemented in compliance with STROBE reporting guidelines, and statistical analyses were conducted using SAS, version 9.4 (SAS Institute) and GraphPad Prism, version 9.2.0 (Dotmatics).

## Results

Of the 12 576 social welfare organizations with Form 990 filings in 2021, 318 (2.5%) were in the health care system. In total, these health care organizations generated revenues of $88.9 billion and profits of $3.2 billion. This represented 70.1% of revenues and 48.8% of profits for all 12 576 organizations combined.

The 318 health care organizations primarily consisted of advocacy groups (n = 106 [33.3%]) and health plans (n = 92 [28.9%]), followed by emergency medical services (n = 44 [13.8%]; such as Belfield Ambulance Service and Sunman Area Life Squad), dental plans (n = 43 [13.5%]), and other groups (n = 33 [10.4%]) (Figure, A). Health plans, dental plans, and advocacy groups combined accounted for almost all of the revenue (98.6%) and profit (99.2%) generated by health care social welfare organizations. The Table summarizes information for the 50 largest health care social welfare organizations (by revenue).

**Figure.**
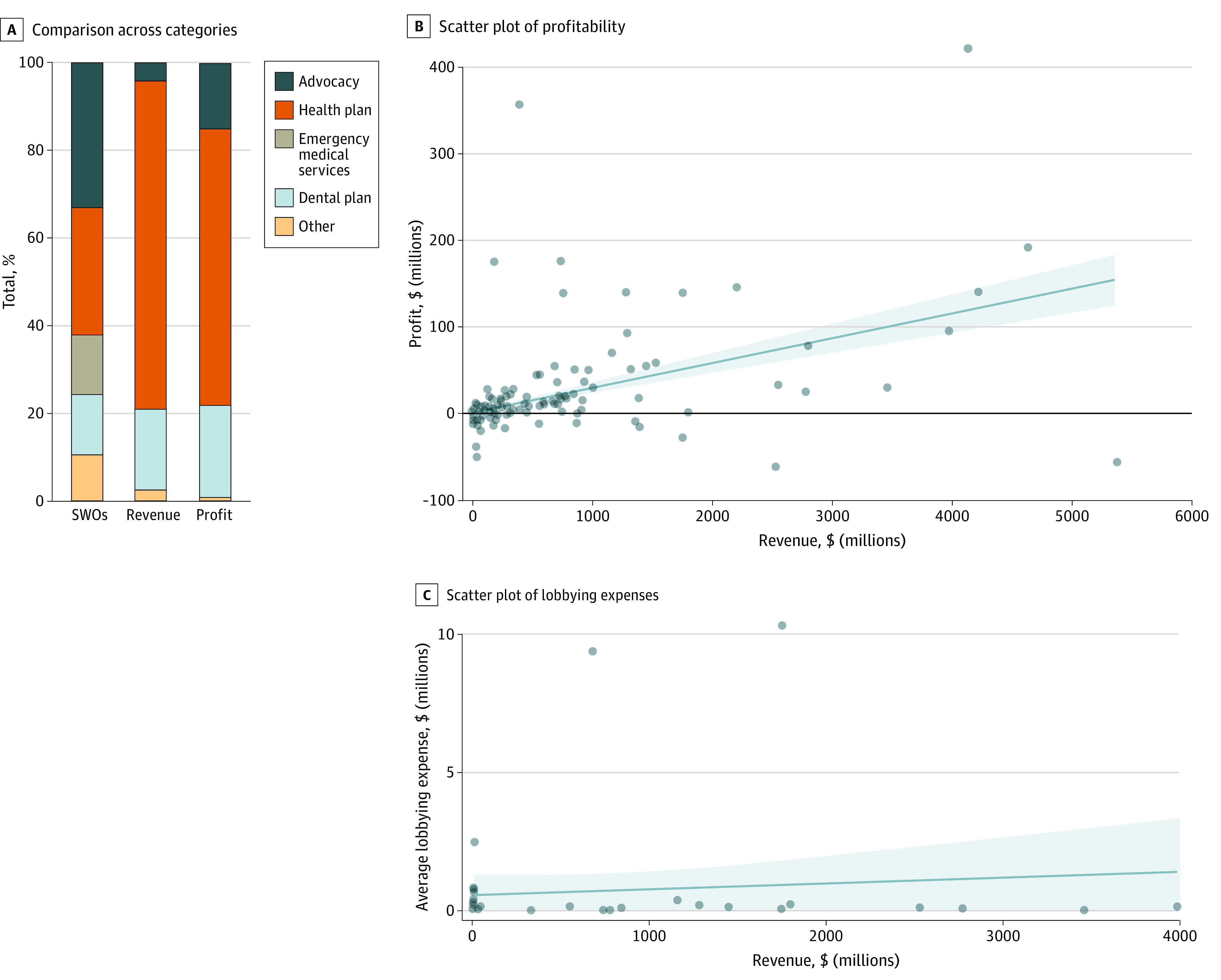
Social Welfare Organizations (SWOs) in the US Health Care System A, The total number of organizations is 318, the total revenue is $88.9 billion, and the total profit is $3.2 billion. B and C, The solid lines represent the line of best fit, and the shaded areas represents the 2-sided 95% CIs. Each dot represents an individual SWO.

**Table.** The 50 Largest Health Care Social Welfare Organizations in the US by Revenue

Rank	Organization name	Category	Revenue, $	Profit margin, %	Average annual lobbying expenses
1	Health Insurance Plan of Greater New York	Health plan	5 381 630 436	−1.0	NA
2	Delta Dental of California	Dental plan	4 636 541 561	4.1	NA
3	Blue Care Network of Michigan	Health plan	4 229 242 702	3.3	NA
4	Priority Health	Health plan	3 987 261 671	2.4	$130 000
5	SelectHealth Inc	Health plan	3 947 746 343	10.7	NA
6	Healthfirst Health Plan Inc	Health plan	3 459 891 700	0.9	$0
7	Tufts Associated Health Maintenance Organization Inc	Health plan	2 805 940 875	2.8	NA
8	MVP Health Plan Inc	Health plan	2 772 334 199	0.9	$65 000
9	HMO Minnesota	Health plan	2 553 100 078	1.3	NA
10	Tufts Health Public Plans Inc	Health plan	2 527 590 380	−2.5	$113 750
11	HealthPartners Inc	Health plan	2 200 384 109	6.6	NA
12	Health Partners Plans Inc	Health plan	1 795 543 344	0.1	$200 000
13	AARP	Advocacy	1 752 770 442	8.0	$10 312 250
14	Capital District Physicians’ Health Plan Inc	Health plan	1 749 273 300	−1.6	$52 500
15	Health Alliance Plan of Michigan	Health plan	1 523 591 589	3.9	NA
16	Independent Health Association Inc	Advocacy	1 443 585 916	3.8	$120 000
17	MDwise Medicaid Network Inc	Health plan	1 393 180 417	−1.1	NA
18	Geisinger Health Plan	Health plan	1 380 938 040	1.2	NA
19	Delta Dental of Washington	Dental plan	1 353 716 791	−0.7	NA
20	MDwise Inc	Health plan	1 319 626 095	3.9	NA
21	Security Health Plan of Wisconsin Inc	Health plan	1 284 857 928	7.2	NA
22	Providence Health Plan	Health plan	1 281 920 151	11.0	$187 500
23	Medica Health Plans	Health plan	1 158 090 888	6.1	$380 000
24	Stratacor	Dental plan	993 667 914	3.1	NA
25	Providence Health Assurance	Health plan	963 717 339	5.2	NA
26	Capital Health Plan Inc	Health plan	926 352 451	4.0	NA
27	Delta Dental Plan of Michigan	Dental plan	913 672 014	1.7	NA
28	Community Health Plan of Washington	Health plan	900 064 466	0.4	NA
29	AstraZeneca Patient Assistance Organization	Other	868 571 806	0.0	NA
30	Allways Health Partners Inc	Health plan	863 119 274	−1.3	NA
31	McLaren Health Plan Inc	Health plan	845 052 646	6.0	$85 000
32	Sharp Health Plan	Health plan	844 861 105	2.6	NA
33	Health New England & Subsidiaries	Health plan	777 982 397	2.2	$0
34	Delta Dental of Illinois	Dental plan	772 477 956	2.6	NA
35	Scott and White Health Plan	Health plan	751 758 378	18.5	NA
36	Western Health Advantage	Health plan	742 178 314	0.2	$0
37	Delta Dental of Pennsylvania	Dental plan	737 749 731	2.3	NA
38	Oregon Dental Service	Dental plan	730 406 810	24.2	NA
39	Delta Dental of Wisconsin Inc	Dental plan	718 772 671	2.9	NA
40	AvMed Inc	Health plan	708 415 108	2.3	NA
41	Community Health Choice Inc	Health plan	706 161 754	1.4	NA
42	The Health Plan of West Virginia Inc	Health plan	703 647 414	5.1	NA
43	Parkland Community Health Plan Inc	Health plan	673 539 980	8.2	NA
44	Blue Cross Blue Shield Association	Health plan	672 909 630	1.8	$9 418 200
45	Delta Dental Plan of Virginia	Dental plan	661 878 422	2.1	NA
46	Delta Dental of New York Inc	Dental plan	590 977 089	1.9	NA
47	Sutter Health Plan	Health plan	589 661 573	2.2	NA
48	Mount Carmel Health Plan Inc	Health plan	562 028 704	8.0	NA
49	Delta Dental of Missouri	Dental plan	556 272 399	1.5	NA
50	Harvard Pilgrim Health Care of New England Inc	Health plan	548 842 588	−2.2	$137 500

Abbreviation: NA, not available (ie, no lobbying disclosures found).

Median (IQR) profit margin was highest for advocacy groups (7.4% [−7.7% to 24.9%]). Other categories’ median profit margins were lower than 4.3%. Some organizations had substantially high profitability (Figure, B). Among 41 health care social welfare organizations that reported lobbying expenses between 2017 and 2020, most spending averaged less than $1 million annually, with 3 exceptions (Figure, C).

## Discussion

Using 2021 IRS filings, we found that social welfare organizations in the US health care system generated more than two-thirds of the revenues and almost half of the profits of all social welfare organizations combined. Profitability and lobbying expenditures varied widely across the organizations. Due to the lack of data, we were unable to identify organizations’ religious affiliations (if any) or other characteristics. Results are subject to potential inaccuracies in administrative data sets.

Social welfare organizations receive substantial subsidies from federal, state, and local taxpayers. They have minimal IRS reporting requirements and oversight. They are also not subject to the Federal Election Commission’s campaign finance disclosure requirement. To improve accountability to taxpayers and help ensure compliance with tax-exemption requirements, policy makers should consider designing tests to evaluate whether an organization’s profitability violates §501(c)(4), determine whether an organization’s social welfare benefits exceed its tax-exempt benefits, and increase reporting transparency for political activities.

## Notes

Supplement. Data Sharing Statement
