# Supplementary material for: Financial Characteristics of Nonprofit Social Welfare Organizations in the US Health Care System
Source: JAMA Health Forum. 2023 Jun 23;4(6):e231507. doi: 10.1001/jamahealthforum.2023.1507 (PMC10290247; doi:10.1001/jamahealthforum.2023.1507)
Supplement: Supplement. — Data Sharing Statement [file jamahealthforum-e231507-s001.pdf]

Data Sharing Statement

Plummer. Financial Characteristics of Nonprofit Social Welfare Organizations in the US Health Care System. *JAMA Health Forum*. Published June 23, 2023.
doi:10.1001/jamahealthforum.2023.1507

Data
Data available: No

Additional Information
Explanation for why data not available: The data used in this study are publicly available.
